# Supplementary material for: Meta-Analysis and Gene Set Enrichment Relative to ER Status Reveal Elevated Activity of MYC and E2F in the “Basal” Breast Cancer Subgroup
Source: PLoS One. 2009 Mar 9;4(3):e4710. doi: 10.1371/journal.pone.0004710 (PMC2650420; doi:10.1371/journal.pone.0004710)
Supplement: Dataset S1 — GSEA results from first screen. The file contains a zipped archive of the HTML results of the second GSEA screen. Reports for enriched or depleted datasets may be accessed from the “index.html” link within the my_analysis.GseaPreranked.1219975950625.rpt.zip ZIP archive. Enrichment plots are available for each of the 50 most enriched and depleted gene sets. (13.32 MB ZIP) [file pone.0004710.s001.zip › my_analysis.GseaPreranked.1219975950625.rpt.zip!//CHR5Q11.html]

Details for gene set CHR5Q11[GSEA]

|  || Dataset | zSco.ERneg.to.ERpos\_collapsed |
| Phenotype | NoPhenotypeAvailable |
| Upregulated in class | na\_neg |
| GeneSet | CHR5Q11 |
| Enrichment Score (ES) | -0.6146633 |
| Normalized Enrichment Score (NES) | -2.0392706 |
| Nominal p-value | 0.0 |
| FDR q-value | 0.0074419677 |
| FWER p-Value | 0.133 |
Table: GSEA Results Summary

  

Fig 1: Enrichment plot: CHR5Q11      
 Profile of the Running ES Score & Positions of GeneSet Members on the Rank Ordered List

  

| PROBE | GENE SYMBOL | GENE\_TITLE | RANK IN GENE LIST | RANK METRIC SCORE | RUNNING ES | CORE ENRICHMENT || 1 | ESM1 |  |  | 2326 | 2.308 | -0.1215 | No |
| 2 | GZMA |  |  | 2627 | 2.130 | -0.0948 | No |
| 3 | RNASEN |  |  | 4723 | 1.246 | -0.2235 | No |
| 4 | HTR1A |  |  | 5384 | 1.019 | -0.2496 | No |
| 5 | PARP8 |  |  | 5925 | 0.840 | -0.2708 | No |
| 6 | ANKRD55 |  |  | 6670 | 0.599 | -0.3129 | No |
| 7 | ISL1 |  |  | 6784 | 0.558 | -0.3085 | No |
| 8 | GZMK |  |  | 7833 | 0.176 | -0.3832 | No |
| 9 | HSPB3 |  |  | 8122 | 0.065 | -0.4034 | No |
| 10 | PELO |  |  | 8139 | 0.060 | -0.4032 | No |
| 11 | FST |  |  | 8866 | -0.240 | -0.4522 | No |
| 12 | MSH3 |  |  | 11027 | -1.592 | -0.5778 | Yes |
| 13 | PPAP2A |  |  | 11478 | -2.009 | -0.5652 | Yes |
| 14 | SKIV2L2 |  |  | 11510 | -2.048 | -0.5202 | Yes |
| 15 | MAP3K1 |  |  | 11996 | -2.557 | -0.4975 | Yes |
| 16 | NDUFS4 |  |  | 12345 | -3.017 | -0.4540 | Yes |
| 17 | DHX29 |  |  | 12353 | -3.025 | -0.3845 | Yes |
| 18 | SFRS12 |  |  | 12641 | -3.513 | -0.3249 | Yes |
| 19 | TAF9 |  |  | 12778 | -3.813 | -0.2469 | Yes |
| 20 | IL6ST |  |  | 12805 | -3.905 | -0.1586 | Yes |
| 21 | MRPS30 |  |  | 12895 | -4.143 | -0.0695 | Yes |
| 22 | MOCS2 |  |  | 12968 | -4.383 | 0.0265 | Yes |
Table: GSEA details [plain text format]

  

Fig 2: CHR5Q11: Random ES distribution      
 Gene set null distribution of ES for **CHR5Q11**
